# Microfluidic Bio-Sensors and Their Applications

**DOI:** 10.3390/bios13090843

**Published:** 2023-08-24

**Authors:** Krishna Kant

**Affiliations:** 1 Biomedical Research Center (CINBIO), University of Vigo, 36310 Vigo, Spain; krishna.kant@uvigo.gal; 2 Centre for Interdisciplinary Research and Innovation (CIDRI), University of Pertoleum and Energy Studies, Dehradun 248007, India

Biosensors are a promising tool for a wide variety of target analyte detection and enable point-of-care diagnostics with reduced volume and space. The integration of sensing elements into microfluidic systems has attained great interest in research. Due to the advanced manufacturing and availability of sensor systems, they have become very cost-effective to produce. Improvements in the field of designing and fabricating microfluidic chips have been significant in recent years. The use of microfluidic systems with integrated sensors have been developed by various new cost-effective approaches like 3D-printed microfluidics, polydimethylsiloxane (PDMS) soft lithography, and laser cutting and micro-milling. These microfluidic chips have been used with various technologies like electrochemical, optical, fluorescence, etc., for their sensing application. These techniques have also forced researchers to alter these microfluidic sensor systems according to changing experimental requirements in a simple and cost-effective manner. Recently, the enhancement of wearable microfluidic- and smartphone-based sensors has become a developing research field for promising widespread point-of-care testing. However, microfluidic chips and developed applications remain in their initial development phases, and they have not yet been largely commercialized. Microfluidic and biosensor advancements are growing hand in hand, both in research and product development, and thus their miniaturization for use in commercial products is also taking place. Microfluidic technology and integrated biosensing represent future goals in this area. Investigating the integration of artificial intelligence and electronics sensor with biological elements to better understand and mimic natural systems. This will eventually enable the development of new products for mankind and society. This Special Issue covers the dedicated innovations across a variety of topics in this area, from sensing to manufacturing and implementation methods to novel microfluidic-based sensors for biological purposes. Researchers have reported on the latest developments in microfluidic-based sensing in glucose, graphene application and applications for cancer cells. The multiplexed sensors and other types of integrated sensor including electrochemical, optical, magnetic, and other transduction types with microfluidics chips are highlighted for the interest for early career researchers.

This Special Issue contains ten excellent papers (eight research articles and two review papers). The research articles focus on the microfluidic-assisted synthesis of metal organic frameworks (MOFs) for sustained drug delivery [1]. It explores the high surface area and addition of functional surfaces to make them ideal drug delivery vehicles. The first article concerns glucose biosensor, presenting the quantification, excellent linearity, temperature calibration, and real-time detection using a resistor and capacitor, combined with a PDMS microfluidic channel [2]. Another article presents an RFID-based glucose microwave biosensor and achieves a non-contact measurement of the concentration of glucose [3]. These studies present innovative applications of microfluidic and biosensors for glucose. Similarly, a graphene oxide-based electrode is presented, promising low-cost and easy fabrication of sensors sensitive for selective label-free DNA biosensing [4]. 

One of the research articles presents integrated microfluidic sensors for rapid analyte detection on antibody-decorated beads for two acute kidney injury biomarkers [5]. A novel silicon-based lab-on-chip sensor to perform low-density and high-resolution multi-assay analysis for DNA amplification and hybridization is also presented to complement this Special Issue [6]. In the study ‘Surfactant-Based Microdroplets and Monitoring Bacterial Gas Metabolism with Droplet-Based Microfluidics’, the authors confirmed that gas cross reaction took place between droplets formed by fluorinated oil and the PFPE–PEG–PFPE surfactant [7]. This allowed the researchers to choose the substrate and material accordingly to avoid any cross reactivity. A further application of microfluidic chips has been demonstrated in cellular study, where an optically induced dielectrophoresis technique was used to alter the cell microenvironment and functionalities [8]. The published review articles focus upon the field of cancer detection. Two different approaches (paper-based and microfluidic channel-based) are discussed, including their advancements in cancer biomarker detection and in capturing CTC cells [9,10]. 

With the collection of papers in this Special Issue, we have taken a step towards the advancement of new microfluidic devices for various microfluidic-based biosensing applications.
